# The Chemical and Evolutionary Ecology of Tetrodotoxin (TTX) Toxicity in Terrestrial Vertebrates

**DOI:** 10.3390/md8030577

**Published:** 2010-03-10

**Authors:** Charles T. Hanifin

**Affiliations:** Hopkins Marine Station of Stanford University, 120 Oceanview Blvd. Pacific Grove CA, 93950, USA; E-Mail: charlesh@stanford.edu; Tel.: +01-1-831-655-6220

**Keywords:** tetrodotoxin, TTX, Amphibia, Caudata, Anura, Salamandridae, Taricha, Notophthalmus, Cynops, Atelopus

## Abstract

Tetrodotoxin (TTX) is widely distributed in marine taxa, however in terrestrial taxa it is limited to a single class of vertebrates (Amphibia). Tetrodotoxin present in the skin and eggs of TTX-bearing amphibians primarily serves as an antipredator defense and these taxa have provided excellent models for the study of the evolution and chemical ecology of TTX toxicity. The origin of TTX present in terrestrial vertebrates is controversial. In marine organisms the accepted hypothesis is that the TTX present in metazoans results from either dietary uptake of bacterially produced TTX or symbiosis with TTX producing bacteria, but this hypothesis may not be applicable to TTX-bearing amphibians. Here I review the taxonomic distribution and evolutionary ecology of TTX in amphibians with some attention to the origin of TTX present in these taxa.

## 1. Introduction

One of the most intriguing natural toxins isolated and described in the twentieth century is the neurotoxin tetrodotoxin (TTX), a non-proteinaceous, low molecular weight toxin (M.W. = 319.3) with extremely high potency (Human LD50 = 10.2 μg/kg). Interest in TTX results from a number of striking circumstances, most significant of these is the vast array of taxa that are now known to possess TTX [1–3]. The presence of TTX in such a wide and disparate array of taxa has been taken as evidence that the ultimate origin of TTX in metazoans must be exogenous and there is good evidence that uptake of bacterially produced TTX is an important component of TTX toxicity in TTX-bearing marine metazoans [2–6]. However, this model has been questioned in regards to the TTX present in terrestrial taxa [1,7–10]. Arguments against bacterially sourced TTX in terrestrial metazoans derive, in part, from two major lines of evidence: (1) unlike marine species in which TTX is found in a wide array of taxonomic groups, TTX in terrestrial metazoans appears to be limited to a single class of vertebrates (Amphibia) with limited distribution within this class, and (2) the presence of multiple analogs of TTX (or saxitoxin (STX)) that are present only within a single species (or genus) such as chiriquitoxin (CHTX) and zetekitoxin (ZTX) in *Atelopus* or those (e.g., 6-*epi*TTX) that are common in the TTX profiles of some TTX-bearing amphibians, but are absent, or a very minor components in the TTX profiles of marine taxa or TTX producing bacteria [11–20], see also [1]. This review will focus on the taxonomic distribution and evolutionary ecology of TTX in amphibians as well as a brief discussion of the structure and pharmacology of amphibian specific TTX analogs.

## 2. Background

Tetrodotoxin takes its name from the Teleost fish order Tetrodontiformes from which the toxin was first isolated and described. Tetrodontid fish, which include puffer fish or *fugu*, have long been known to be toxic [21]. In fact, there is evidence that early Egyptians (5^th^ dynasty, ca 2500 BC) were aware of the toxicity associated with these fish [21]. Chinese herbal medical writings from the first or second century BC also describe pharmacological effects associated with the flesh and eggs of these fish [22]. Although little formal research was done on TTX until the late 1800’s, European natural historians were aware of these toxic fish through historical texts (e.g., Kaempfer’s History of Japan, from [22]). The earliest example of formal research into the pharmacology of TTX appears to have been Charles Remy’s work in which he described the symptoms of TTX poisoning and documented the high concentrations of TTX present in the gonads of puffers (Remy, 1883, from [21]). Later work in the 19^th^ century included a comprehensive pharmacology of TTX by Takahashi in 1889, from [21].

Tetrodotoxin was first formally named in 1909 by Tahara, from [23], who prepared a crude extract from puffer fish. Pure, crystalline TTX was not isolated until 1950 when A. Yokoo isolated TTX from the ovaries of *Fugu rubripes* and described it as spheroidine after a genus of puffer fish [23]. The nomenclature of TTX was solidified in 1952 when K. Tsuda and M. Kawamura isolated an identical toxin using chromatographic methods and named it tetrodotoxin (from [23]).

The complete molecular structure of TTX was first described in 1964 at the Natural Products Symposium of the International Union of Pure and Applied Chemistry by a total of 4 different lab groups including K. Tsuda, T. Goto, R. B. Woodward, and H. S. Mosher [21]. It is important to note that while three of these groups had been working on toxin isolated from puffer fish, the Mosher group was reporting on compound they named tarichatoxin isolated from eggs of the newt *Taricha torosa* [24–26]. Since the 1960’s the chemistry, pharmacology, and synthesis of TTX has been the subject of a voluminous body of work, see [27], as well as [1,3,21,23,28–31] for partial reviews.

The pharmacology of TTX is well studied and will not be detailed here, see [27] for recent review. The gross pharmacological effects of TTX (*i.e.*, muscle paralysis and/or death) have long been recognized [22,32–35], also see [21], but it was not until the 1950’s that a more detailed understanding of the pharmacological properties of TTX began to emerge. Tetrodotoxin was shown to block sodium currents in excitable membranes (e.g., nerve and muscle tissue) [36–38]. It is now understood that TTX binds and blocks voltage-gated sodium (Na^+^) channels with remarkably high specificity thereby prevented in the influx of Na^+^. These ion channels are, in part, responsible for the initiation and propagation of action potentials in most metazoans [28,36,39–41]. The current model of the interaction between TTX and voltage-gated Na^+^ channels is that the positively charged amino end of TTX forms complex electrostatic bonds with two charged rings of amino-acid residues in the outer pore of the sodium channel (the selectively filter) [29,30,42]. The remainder of the TTX molecule then blocks the outer pore preventing the influx of Na^+^ ions and the associated currents required for membrane depolarization and action potential initiation.

## 3. TTX and TTX Analogs in Amphibians

**Figure 1:**
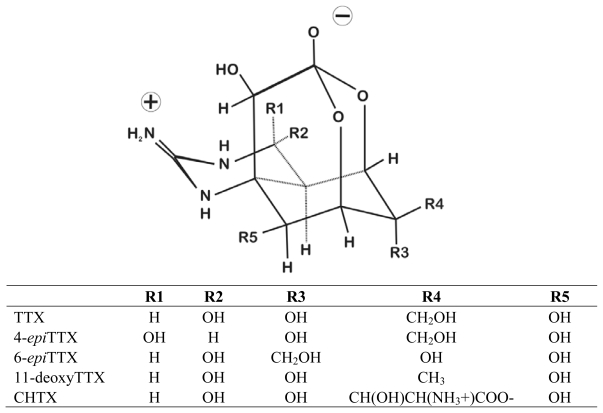
The structure of TTX as well as some TTX analogs associated with amphibians (from Yostu-Yamashita 2001) [15].

Tetrodotoxin is a guanidium ion with a complex oxygenated cyclohexane framework with both guanidine and ortho-acid functional groups (Figure 1) [27,43,44]. Numerous natural, semi-synthetic, and synthetic analogs of TTX have been reported, reviewed in [15]. A detailed review of these TTX analogs is beyond the scope of this review, but these analogs can be broadly grouped as either hemilactal, or lactone variants [15,44]. Amphibians have been an especially plentiful source of TTX analogs [11,12,14,16,19,45–47]. The hemilactal forms of TTX are the more common naturally occurring analogs (Figure 1). Many of the commonly seen analogs (e.g., 4-*epi*TTX and anhyrdoTTX) are likely conversion or equilibrium products of TTX and are commonly seen in all TTX-bearing taxa [44]. As such they are of interest to biochemists and may shed light on the synthesis of TTX but may not be informative in regards to possible differences in the TTX toxicity of marine versus terrestrial taxa. However, other analogs (e.g., CHTX, Figure 1) appear to be present only in amphibians and restricted to one or two closely related species (e.g., CHTX and ZTX are found only in the toad genus *Atelopus*) [16,19,46,48]. These analogs are extremely potent and have toxicities equivalent or greater than TTX itself [11,14,47,49]. Understanding their distribution and origin are critically important in the exploration of TTX in Amphibians. The unique structure of these analogs does not inherently support an endogenous origin of TTX in amphibians. Multiple alkaloid toxins unique to species or genera of dendrobatid frogs are now known to come from precursors present in arthropod prey of these frogs [50]. It is possible that the presence of CHTX and ZTX in *Atelopus* results from similar processes but there is little evidence that supports this hypothesis (but see, [19] for recent work).

Other analogs present in amphibians are not as potent as TTX (e.g., 6-*epi*TTX, Figure 1), but are of interest because they do not appear to be cross convertible with TTX and appear to form as a result of stereo-specific reactions [12, 44]. One of these analogs (6-*epi*TTX) was first described in the newt genus *Cynops* [12]. This analog can represent a significant portion of the total TTX present in TTX-bearing salamanders but appears to be relatively rare in marine taxa [12,13,17,18,51–53]. In populations of *Taricha* the relative levels of TTX to 6-*epi*TTX are invariant within a population, but display significant variation among population [54]. Similar patterns have also been documented in populations of *Cynops pyrrhogaster* in Japan [55]. In *Taricha* this variation in toxin profiles can occur over very short distances (<20 km) and among populations that occupy the same watershed as well as similar habitats (unpublished data). Spatial variation in analog profiles has been seen as an additional argument in favor on an exogenous origin for TTX in metazoans [3], but can also be seen to favor an endogenous origin if seen in the context of generic variation associated with the genes that comprise the biosynthetic pathway of TTX amphibians versus marine bacteria.

## 4. Taxonomic Distribution of TTX and TTX Analogs in Amphibians

Although TTX is broadly distributed across taxonomic classes in aquatic species (reviewed in [3]), in terrestrial taxa TTX appears to be limited to two orders (Anura, and Caudata) of a single class (Amphibia) of vertebrates [1]. Tetrodotoxin (or TTX analogs) have been identified in a total of 28 species representing 10 genera and six (or five, see below) families (Anura: Bufonidae, Rhacophoridae, Brachycephalidae, Dendrobatidae; Caudata: Ambystomatidae, Salamandridae) (Table 1), but appear to be absent in a total of 38 examined species (Table 2), reviewed in [1].

The earliest confirmation of TTX in amphibians was in eggs of the California Newt *Taricha torosa* (Order: Caudata, Family: Salamandridae) [24–26]. However, the presence of a neurotoxin in skin and flesh of this species that had functional similarities to TTX had been known since the 1930’s [33–35,56,57]. The discovery of TTX, the TTX analog CHTX and the saxitoxin analog ZTX in the toad genus *Atelopus* (Order: Anura, Family: Bufonidae) was the first evidence of TTX in a non-salamandrid [46], but see also [58] for earlier work.

Since Daly’s review [1], TTX has been identified (or confirmed) in two additional species of *Brachycephalus* (*B. ephippium*, and *B. pernix* [53]), four species of the European newt genus *Triturus* (*Tr. vulgaris*, *Tr. alpestris*, *Tr. cristatus*, and *Tr. helveticus*, [18]), three species of *Atelopus* (*A. varius*, *A. chiriquiensis*, and *A. zeteki*, [19]) (Table 1). Recent work has also confirmed the presence of 6-*epi*TTX and/or 11-*oxo*TTX in *Triturus* [18] and *Brachycephalus* [53] as well as the presence of CHTX in an Atelopid species other than *A. chiriquiensis* (e.g., *A. limosus* and *A. glyphus* [19]) (Table 1).

The identification of TTX in two families of Caudata is somewhat problematic. Although TTX has been identified in both the Salamandridae and Ambystomatidae (Table 1), I would argue that in Caudates, TTX is likely limited to a subset of related genera (*Taricha*, *Notophthalmus*, *Triturus*, *Cynops*, and *Paramesotriton*) in the family Salamandridae (see also [1]) and that reports of the presence of TTX in the Ambystomatidae are likely erroneous. With the exception of two reports (both apparently based on results from the same specimen) of TTX in *Ambystoma tigrinum* [12,13], there is no evidence of the presence of TTX in a non-salamandrid Caudate (Table 1, Table 2). Earlier investigations specifically examined *A. tigrinum* for the presence of TTX and did not detect any evidence of TTX of a TTX-like toxin [59] nor did an additional examination of a single *Ambystoma tigrinum* using HPLC-FLD by this author (unpublished data). More significantly, species of *Ambystoma* are highly sensitive to TTX [26,33,57,63], yet other TTX-bearing salamanders (as well as other TTX-bearing vertebrates) are highly resistant to TTX [22,25,26,32,60,63,77,78]. Given that the report of TTX in this species is based on a single animal with questionable provenance [12], a reexamination of the presence of TTX in *Ambystoma* seems to be in order.

## 5. Ecology and Evolution of TTX Toxicity in Amphibians

The ecological role of TTX in metazoans (both marine and terrestrial) is of critical importance, yet it is understudied and poorly understood. Bioaccumulation of TTX (whether through synthesis, symbiosis with bacteria, or dietary uptake and processing) likely results in a significant cost to TTX-bearing taxa [79]. Additionally, there is evidence that TTX resistance (a necessary trait for accumulation of TTX in TTX-bearing vertebrates) may also come with its own cost [29,80,81]. As a result, metazoans that possess TTX must gain some benefit from their TTX toxicity that outweighs these costs [79].

Amphibians are present on every continent except for Antarctica and occupy a diverse array of habitats [82]. Their slow speed, soft bodies, and habitat choices make them attractive targets of predators. In response, amphibian species worldwide have evolved a pharmacopeia of toxic and noxious compounds [83]. These compounds protect amphibians from predation [7,84–87] as well as infection from pathogens (both fungal and bacterial) [88–90]. The TTX present in TTX-bearing amphibians is also assumed to play a defensive role and there is good data to support the hypothesis [1,83].

The presence of TTX is associated with aposematic coloration in TTX-bearing amphibians. Species of *Atelopus*, *Brachycephalus*, and *Colostethus* that possess TTX (or CHTX) also possess aposematic coloration, but non-TTX bearing species of *Brachycephalus* do not appear to have TTX [7,53,83]. In one study aposematic efts of *Notophthalmus* were found to be more toxic than non-aposematic adults [59], but another found the opposite pattern [51]. Salamandrid newts are well known for an array of defensive warning postures [84,91]. TTX-bearing salamandrid newts (e.g., *Taricha*, *Cynops*, and *Notophthalmus*) engage in a well-documented warning pose in which they display orange, red, or yellow present on their ventral surface [92]. In *Taricha*, at least, this warning posture is frequently associated with secretion from dorsal glands that contain TTX (pers. obs.).

Defensive compounds of amphibians are typically associated with secretory skin glands [82,84,93–97]. In TTX-bearing amphibians levels of TTX are typically much higher in skin than in other tissues (except for eggs see below) [8,13,17,18,20,46,53,59,64]. Furthermore there is direct evidence than TTX is contained and sequestered in granular skin glands of TTX-bearing salamanders [61,64,98]. *Taricha* and *Cynops* newts actively secrete TTX when directly stimulated or when they encounter a snake predator [8,61,99]. Skin secretions from *Taricha* [25,60,63,100–102], and *Notophthalmus* [103–109], are known to be lethal to (or deter predation by) potential predators. Reports associated with the toxicity of *Polypedates* also indicate that secretions from this species appear to be toxic to potential predators [76]. These results are not surprising given the high levels (and potency) of TTX present in these taxa are further support for a defensive role of TTX in amphibians.

The best-documented example of the defensive role of TTX is the coevolutionary interaction between *Taricha* newts and snake predators. Newts of the genus *Taricha* have long been known to possess a TTX resistant predator (garter snakes of the genus *Thamnophis*) [56,57,63]. In some populations that co-occur with newts, garter snakes have evolved resistance to TTX allowing them to prey on toxic newts. This predation by snakes has generated a coevolutionary arms race centered on TTX levels in newts and TTX-resistance in garter snakes [67,110–113]. Although the strength of coevolution between these species is spatially variable, these arms races have generated elevated (and extreme) levels of TTX and TTX resistance in some populations of *Taricha* and *Thamnophis* [54,64,67,110,111,113–116]. Tetrodotoxin levels present in *Taricha* (and other TTX-bearing Salamandridae) are significantly higher than TTX-bearing anurans (Table 1, also see [1]). Individuals from some populations of *Taricha granulosa* have been measured to possess up to 14 mg of TTX [67]. In *Taricha*, these elevated levels of TTX result from coevolution with garter snakes [67,111]. There is no direct evidence that similar predator-prey interactions have driven elevated TTX levels in other species of Salamandridae, but populations of both *Triturus* in Germany and *Cynops* in Japan show patterns of spatial variability in TTX levels [18,55] and environmental factors alone seem an unlikely explanation for these differences or the elevated levels of TTX seen in salamanders.

Tetrodotoxin also serves to defend the eggs of TTX-bearing amphibian species. Ecologically relevant levels of TTX have been found in the eggs of TTX bearing salamanders and *Atelopus* [1,25,46,59,65,71,100]. In *Taricha*, individual eggs can possess upwards of 2 μg of TTX and the investment of TTX in eggs appears to be an active process [65]. Recent evidence indicates that Caddis Fly larva are possible predators of *Taricha* eggs and that increased levels of TTX in the eggs of newts may deter predation by these insect predators [117].

The presence of TTX in adult newts (and possibly in Anurans) has far reaching ripple effects in the communities in which these animals exist [118]. There are at least two mimicry systems associated with the presence of TTX in N. American newt genera (*Taricha* and *Notophthalmus*, [119–122]). Developing evidence suggests that garter snake predators of newts may be capable of sequestering TTX obtained from newts as defense against their own predators [79, 99]. Tetrodotoxin also seems to serve as warning chemical; allowing larval *Taricha* to sense and avoid cannibalistic predation by adults [118,123,124].

## 6. Conclusions in Regards to the Origin/Biosynthesis of TTX in Amphibians

The ultimate origin or biosynthesis of TTX in amphibians is still a source of some controversy [1,3,8,18,19,67,125]. A compelling argument has been made that TTX present in marine metazoans is derived from bacterial sources (reviewed in [3] but see [126]). However little progress has been made in directly elucidating the genes and enzymatic pathways responsible for the biosynthesis of TTX in bacteria. Studies of TTX biosynthesis in amphibians are mixed and evidence favoring an endogenous origin of TTX in these taxa is indirect at best. In the only study to directly look at TTX synthesis in *Taricha*, animals fed a series of radioactive-labeled (potential) TTX precursors and small molecules did not show evidence of uptake of the radioactive-label [66]. Adult *Atelopus varius* raised in captivity did not possess measurable levels of TTX or TTX like toxins nor did captive *Cynops pyrrhogaster* [3,74]. However *Taricha granulosa* kept in captivity and feed earthworms were capable of maintaining (or increasing) high levels of TTX over multiple years and captive *T*. g*ranulosa* also regenerate large amounts (up to 3 mg) of TTX in relatively brief periods when fed a non-toxic diet [8,9,45]. Similar maintenance of TTX levels over 3 years in captivity has been seen in *Atelopus oxyrhynchus* [48] An examination of the skin and glands of *T. granulosa* did not yield any evidence of symbiotic TTX producing bacteria in granular secretory glands associated with TTX in salamanders [10]. More compelling, perhaps, is the limited distribution of TTX analogs in various species of amphibians [1,7–9,83]. Chiriquitoxin and ZTX have only been described in species of *Atelopus* [1,19]. The 6-*epi*TTX stereoisomer of TTX is common in amphibians and can make up a substantive portion of the total TTX’s present in some species (and/or populations) [12,13,17,18,44,54], yet it appears to be very rare in marine species. In *Taricha* the ratio of 6-*epi*TTX to total TTX show little (or no variation) within a locality, but can vary dramatically across localities; a pattern that is difficult to understand in the context of a dietary or symbiotic source of TTX [54]. Finally, evidence from 40 years of study coevolution between *Taricha* and *Thamnophis* strongly suggest that the elevated (and extreme) levels of TTX seen in the genus likely results from coevolution with snakes and that the evolution of extreme toxicity may have occurred over a relatively short time frame [67,111,127]. These results suggest, in turn, that TTX levels in this genus (and possibly in other salamanders or amphibians) are (to some degree) under genetic control.

## 7. Future Directions

A century after its formal naming interest in TTX is still strong and the molecule is still the focus of extensive research. Although much progress has been made, fundamental questions associated with the synthesis and taxonomic distribution of TTX still remain. The central question facing workers interested in the chemical ecology and evolutionary biology of TTX toxicity in amphibians is still that of an endogenous versus exogenous origin of TTX. Compelling, yet indirect, evidence exists for either position. Any convincing resolution to this problem will have to address the following issues: (1) The presence of TTX analogs that are limited to a single species (or genus) of amphibian, (2) the apparent abundance of TTX analogs such as 6-*epi*TTX and 11-deoxyTTX in the toxin profiles of TTX-bearing amphibians and their corresponding paucity in marine taxa, (3) the extreme spatial variation in toxin profiles and TTX levels seen within populations of some terrestrial TTX-bearing species, (4) the longevity of TTX in (some) captive TTX-bearing species, and (5) the extremely limited taxonomic distribution of TTX in terrestrial vertebrates.

## Figures and Tables

**Table 1:** Distribution and levels of TTX and TTX analogs in amphibians.

Order	Family, Species	Primary Toxin	Estimated amount of TTX (or equivalents) per individual (ug)	Additional Analogs	References
Caudata	**Ambystomatidae**				
	*Ambystoma tigrinum*	TTX	12.6–17.6	6-epiTTX, 11-deoxyTTX	[13]
	**Salamandridae**				
	*Cynops ensicauda*	TTX	9.6–1540	6-epiTTX, 11-deoxyTTX	[12,13,26,59]
	*Cynops pyrrhogaster*	TTX	8–616	6-epiTTX, 11-deoxyTTX	[13,26,55,59–61]
	*Notophthalmus viridescens*	TTX	9.6–220	6-epiTTX, 11-deoxyTTX	[17,18,26,51,59,60,62]
	*Paramesotriton hongkongensis*	TTX	8–11		[13,60]
	*Taricha granulosa*	TTX	<1–14,000	6-epiTTX, 11-deoxyTTX	[8,9,13,24–26,45,54,59,60,63–67]
	*Taricha rivularis*	TTX	96–550		[56,59,60,68]
	*Taricha torosa*	TTX	<1–3000	6-epiTTX	[59,60,66,67]
	*Triturus alpestris*	TTX	0–41	6-epiTTX	[13,18,59]
	*Triturus cristatus*	TTX	0–9	6-epiTTX	[18,59]
	*Triturus helveticus*	TTX	0–8	6-epiTTX	[18]
	*Triturus marmoratus*	TTX	0.16–0.66		[26,59]
	*Triturus vulgaris*	TTX	0–8	6-epiTTX, 11-deoxyTTX	[13,18,59]
Anura	**Brachycephalidae**				
	*Brachycephalus ephippium*	TTX	<1–22.4	6-epiTTX, 11-deoxyTTX	[20,52,53,69]
	*Brachycephalus pernix*	TTX	5		[53]
	**Dendrobatidae**				
	*Colostethus inquinalis*	TTX	0.1–1.4		[70]
	**Bufonidae**				
	*Atelopus chiriquiensis*	CHTX	33 (TTX), 77 (CHTX)	TTX	[14,19,46,47,71]
	*Atelopus glyphus*	CHTX	34–79		[19]
	*Atelopus ignescens*	TTX	<1.0–1.5		[70]
	*Atelopus limosus*	CHTX	8–19		[19]
	*Atelopus oxyrhynchus*	TTX	32–198		[48,72]
	*Atelopus peruensis*	TTX	3.2–4.4		[73]
	*Atelopus spumarius*	TTX	1.6–3.5		[70]
	*Atelopus spurelli*	TTX	<1–1.1		[70]
	*Atelopus subornatus*	TTX	3.2–17.6		[73]
	*Atelopus varius*	TTX	16–26		[19,46,70,74]
	*Atelopus zeteki*	ZTX	<1–264		[19,46,58,70,75]
	**Rhacophoridae**				
	*Polypedates sp.*	TTX	4.8–198		[76]

a Estimates of per individual TTX in μg are based, in part, on conversion from mouse units (MU) taken from Daly 2004 [1]. A mouse unit corresponds to 0.16–0.22 μg of TTX. CHTX and ZTX are based on TTX equivalents.

b Only the presence of 6-*epi*TTX and 11-deoxyTTX are identified here for additional congeners see Daly 2004 [1].

**Table 2:** Species of Amphibians that do not appear to possess TTX.

Order	Family, *Species*	Reference
Caudata	**Ambystomatidae**	
	*Ambystoma tigrinum*	[59]
	**Amphiumadae**	
	*Amphiuma means*	[59]
	*Cryptobranchidae*	
	*Cryptobranchus alleganiensis* *Plethidontidae*	[59]
	*Batrachoseps attenuatus*	[59]
	*Ensatina eschscholtzi*	[59]
	*Aneides lugubris*	[59]
	**Proteidae**	
	*Necturus maculosus*	[59]
	**Salamandridae**	
	*Salamandra salamandra*	[59]
	*Echinotriton andersoni*	From Miyazawa, 2001[2]
Anura	**Bufonidae**	
	*Atelopus certus*	[19]
	*Bufo boreas*	[59]
	*Bufo regularis*	[70]
	*Dendrophyryniscus minutus*	[70,73]
	*Melanophryniscus moreirae*	[70]
	*Melanophrynicus stelzneri*	[73]
	*Oreophrynella sp.*	[73]
	**Denrobatidae**	
	*Aromabates nocturnus*	[70]
	*Dendrobates pumilio*	[70]
	*Phyllobates bicolor*	[70]
	**Hylidae**	
	*Cyclorana australis*	[70]
	*Hemiphractus fasciatus*	[70]
	*Hyla cinera*	[59]
	*Litoria albuguttata*	[70]
	*Nyctimystes tympanocryptis*	[70]
	*Osteocephalus taurinus*	[70]
	*Phrynohyas venulosa* *Leptodactylidae*	[70]
	*Eleutherodactylus gollmeri* *Microhylidae*	[70]
	*Otophryne robusta*	[70]
	*Phrynomerus bifasciatus*	[70]
	*Scaphiophryne marmorata*	[70]
	**Mantellidae**	
	*Mantella aurantiaca* *Myobatrachidae*	[70]
	*Heleioporus albopunctatus*	[70]
	*Notaden nichollsi*	[70]
	*Pseudophryne corroboree*	[70]
	**Pipidae**	
	*Xenopus laevis*	[59]
	**Ranidae**	
	*Rana pipiens*	[59]
	*Rana rugulosa*	[70]
	*Rana septentrionalis*	[70]
